# Aesthetics and mental health: an increase in personality disorders

**DOI:** 10.1192/j.eurpsy.2022.1706

**Published:** 2022-09-01

**Authors:** C. Garcia-Bernal, G. Rodriguez-Menéndez

**Affiliations:** 1 University Hospital Virgen del Rocio, Psychiatry, Seville, Spain; 2 University Hospital Virgen Macarena, Psichiatry, Seville, Spain

**Keywords:** Aesthetics, personality disorder, dysmorphophobia

## Abstract

**Introduction:**

There has been a growing interest in our society for aesthetic interventions and achieving perfect beauty standards. We analyze its relationship with the mental health of our present time.

**Objectives:**

1. Describe the most frequent pathologies associated with aesthetic interventions.

2. Describe the population that most frequently uses these interventions.

3. Management of this pathology.

**Methods:**

Systematic bibliographic review of the literature of the last 5 years following the PRISMA recommendations between March and June 2021.

**Results:**

4 articles were included. Most of them coincide in a high prevalence of borderline personality disorders, high impulsivity, high levels of anxiety, low perceived self-esteem and dysmorphophobia. Greater coordination between physicians who are dedicated to aesthetics and mental health is proposed due to the rise of this fashion.

**Conclusions:**

1. High increase in the use of aesthetic techniques.

2. Women who consume these techniques more.

3. High prevalence of personality disorders.

4. High prevalence of dysmorphophobia.

5. Referral is recommended in some cases to mental health consultations for specific treatment.

**Disclosure:**

No significant relationships.

